# Quantifying the Piezoresistive Mechanism in High-Performance Printed Graphene Strain Sensors

**DOI:** 10.1021/acsami.1c21623

**Published:** 2022-01-31

**Authors:** Eoin Caffrey, James R. Garcia, Domhnall O’Suilleabhain, Cian Gabbett, Tian Carey, Jonathan N. Coleman

**Affiliations:** School of Physics, CRANN & AMBER Research Centres, Trinity College Dublin, Dublin D2, Ireland

**Keywords:** electromechanical, network, pressure, tunneling, sensing

## Abstract

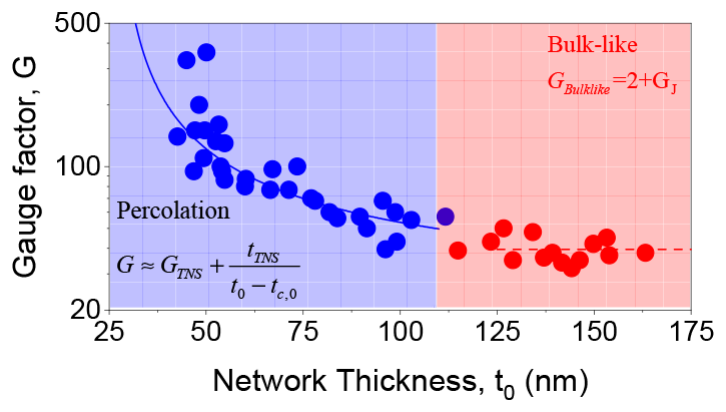

Printed strain sensors will be important in applications such as wearable devices, which monitor breathing and heart function. Such sensors need to combine high sensitivity and low resistance with other factors such as cyclability, low hysteresis, and minimal frequency/strain-rate dependence. Although nanocomposite sensors can display a high gauge factor (*G*), they often perform poorly in the other areas. Recently, evidence has been growing that printed, polymer-free networks of nanoparticles, such as graphene nanosheets, display very good all-round sensing performance, although the details of the sensing mechanism are poorly understood. Here, we perform a detailed characterization of the thickness dependence of piezoresistive sensors based on printed networks of graphene nanosheets. We find both conductivity and gauge factor to display percolative behavior at low network thickness but bulk-like behavior for networks above ∼100 nm thick. We use percolation theory to derive an equation for gauge factor as a function of network thickness, which well-describes the observed thickness dependence, including the divergence in gauge factor as the percolation threshold is approached. Our analysis shows that the dominant contributor to the sensor performance is not the effect of strain on internanosheet junctions but the strain-induced modification of the network structure. Finally, we find these networks display excellent cyclability, hysteresis, and frequency/strain-rate dependence as well as gauge factors as high as 350.

## Introduction

The rise of nanomaterials has led to a renaissance in sensor development, allowing the detection of a multitude of parameters including pressure,^1^ magnetic fields,^2^ temperature,^3^ as well as the presence of unwanted gases,^4^ ions,^5^ chemicals,^6^ or bacteria.^7^ More recently, growth in the wearable technology industry has seen personal sensors enter our daily lives, for example, providing personalized^8^ real-time health and activity monitoring.^9^ Of particular importance in sensing are electromechanical strain sensors, which detect mechanical deformation, converting strain (or stress/pressure) into a change in electrical properties, typically a change in the sensor resistance.^10^ In such piezoresistive sensors, the sensor sensitivity is expressed via the gauge factor (*G*), which is defined as Δ*R*/*R*_0_ = *G*ε in the limit of low strain (i.e., where the resistance response is linear with strain^10,11^). This parameter is one of the most important and certainly the most studied in piezoresistor research. However, for sensors to be useful, as well as having high *G*, they also need to have a good linear range, low load/unload hysteresis, and minimal variation of *G* with frequency.^12−15^ Ideally, they would also be relatively easy to fabricate and install where needed.^16−18^ In terms of commercial sensors, while metal foil strain gauges are relatively cheap and simple to produce,^10^ these have a relatively low *G*-value of ∼2 as the resistance change is based entirely on geometric changes.^19^

Much effort has been made to develop sensors with gauge factors well beyond *G* ≈ 2. Many researchers have turned to materials science to fabricate sensing materials with high *G*-values while minimizing negative properties such as hysteresis and frequency dependence.^16−18,20^ Nanocomposites have shown great promise due to their versatility and the ability to tune sensor response by varying the matrix, the filler, and the composition^11,21^ with hundreds of papers reporting results for piezoresistive nanocomposites with gauge factors as high as 2600.^22^ However, composite sensors have a number of limitations: for example, the conductivity can be low, partly due to polymer coatings around the conducting filler particles.^23^ In addition, high load/unload hysteresis has been reported in some composites.^13,16^ Particularly in soft composites, hysteresis and frequency/strain-rate dependence have been linked directly to the viscoelasticity of the polymer matrix.^11^

One possible way to address problems associated with the polymer matrix would be to avoid it altogether. In this way, a number of groups have reported systems where the piezoresistive element is simply a network of conductive nanoparticles^24^ (e.g., CNTs,^25^ graphene,^26,27^ gold nanoparticles,^28^ MXenes,^29^ TMDs,^30^ and silver NPs/graphene^31^). A considerable advantage of such systems is that the absence of interparticle polymer coatings results in a network conductivity considerably higher than that found in nanocomposites.^23^ Such networks have the added advantages that they are printable.^13,24,32−34^ As with nanocomposites, the received wisdom is that such networks are piezoresistive due to the effect of stain on interparticle charge transport,^11^ although a variety of mechanisms have been hypothesized for different networks.^15^

Graphene is a particularly important component of nanostructured piezoresistive sensors, both as a conductive filler in nanocomposites,^11^ as well as in (polymer-free) films and networks. Mono- and bilayer graphene sheets have a relatively low intrinsic gauge factor of <10.^35−38^ However, much higher values can be obtained by fabricating nanostructured films consisting of arrays of graphene sheets or nanographene films of weakly coupled grains. In this way, graphene-only piezoresistive films have been fabricated through a range of methods including drop casting,^39^ laser scribing,^40,41^ inkjet printing,^32^ spray coating,^33^ and CVD^26,42,43^ (tabulated in the Supporting Information). In these reports, gauge factors as high as ∼600^42^ were obtained for CVD-grown nanographene films. Similarly, strain sensors based on CVD-grown films^44,45^ and nanocomposites^46^ of other 2D materials have also been demonstrated.

Although networks of nanoparticles in general and graphene in particular have some advantages as electromechanical sensors, their performance tends to be poorly characterized in published works with very little data given about sensor hysteresis or frequency dependence. In addition, the effect of network thickness on electromechanical response has not been quantitatively examined, while the piezoresistive mechanism appears to be very poorly understood, beyond the general assumption that the effect of strain on internanosheet transport is dominant. While attempts have been made to model nanomaterial-based strain sensors,^47,48^ the proposed models are not comprehensive and do not appear to fully consider the effects of strain on both network dimensions and network conductivity. In addition, analysis of the latter contribution should consider all strain-induced changes in conductivity, not just the effect of strain on junction resistance.

Here, we show that printed semitransparent graphene strain sensors can exhibit an extremely high gauge factor while also having low hysteresis, good frequency independence, and cyclability over thousands of cycles. Furthermore, we have developed a model that relates gauge factor to both conductivity and network thickness for percolating networks of nanoparticles, which can hopefully guide future studies toward creating higher gauge factor sensors, through a mechanistic understanding of the piezoresistive effect in these systems.

## Results and Discussion

### Material Production and Characterization

**Figure 1:**
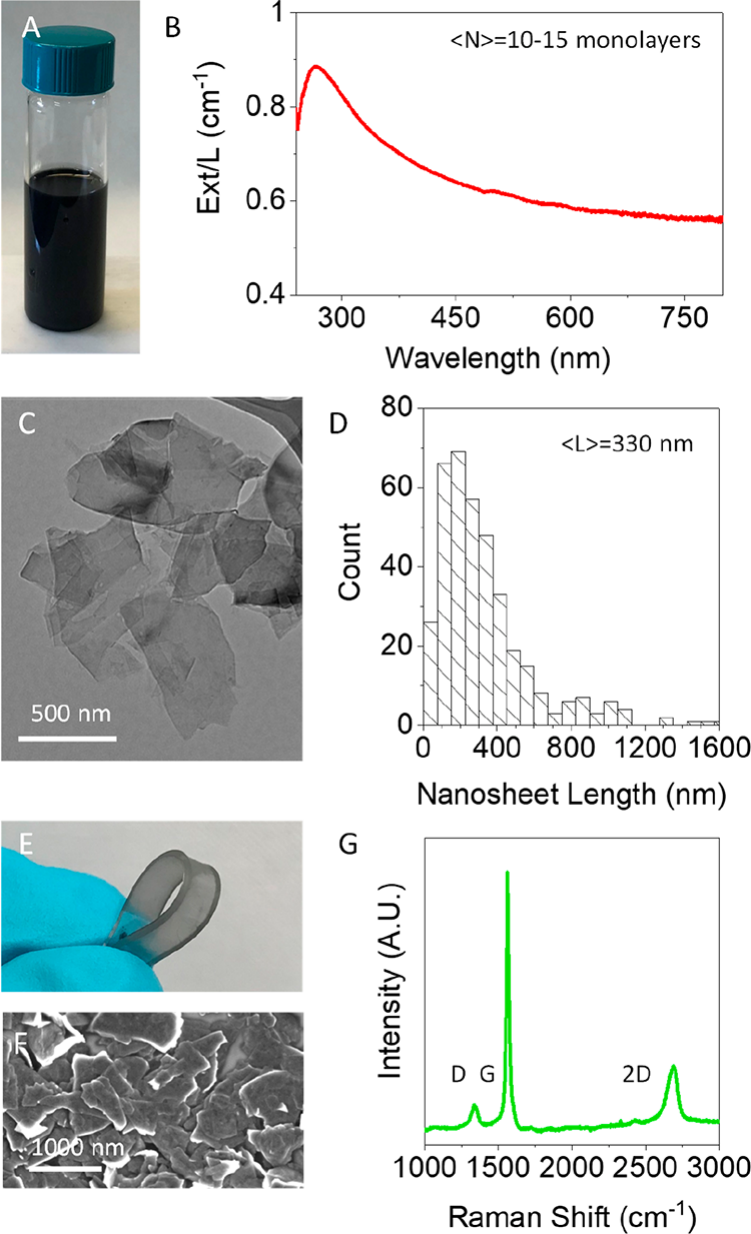
Graphene ink characterization. (A) Image of the graphene/chloroform ink. (B) Extinction spectrum of the ink with the chloroform background removed. As described in the text, this spectrum is consistent with a mean nanosheet thickness of 10–15 monolayers (3–5 nm). (C) Transmission electron microscope (TEM) image of the nanosheets found in the ink. (D) Nanosheet length distribution histogram from TEM images, 376 nanosheets measured, mean length of 330 ± 14 nm. (E) Image of a printed, semitransparent graphene sensor on PDMS substrate. (F) Scanning electron microscope (SEM) image of spray-printed graphene film on PDMS. (G) Raman spectrum of a nanosheet network produced from ink drop cast onto Si/SiO_2_ with the position of the D, G, and 2D bands indicated.

The strain sensors were deposited by spray-casting of a graphene-based ink. The graphene ink was produced by liquid-phase exfoliation (LPE)^49−51^ as described in the Experimental Methods (Figure 1A). A typical extinction spectrum is shown in Figure 1B, along with the characteristic high frequency plateau and graphitic π–π* transition just below 300 nm.^52^ The ink concentration and estimated mean number of monolayers were determined from published metrics^53^ to be *C* = 2 mg/mL and ⟨*N*⟩ ≈ 10–15 layers. A typical transmission electron microscopy (TEM) image is shown in Figure 1C and shows the nanosheets to be irregularly shaped as is typical for those produced by LPE.^50^ TEM images were used to extract the nanosheet length distribution as shown in Figure 1D: sampling 376 nanosheets, the mean nanosheet length was found to be 330 ± 14 nm.

Dispersions such as that in Figure 1A can be used to deposit thin films by spray casting^54^ (Experimental Methods). Figure 1E is an image of a thin, spray cast film deposited on a polydimethylsiloxane (PDMS) substrate. It has been bent back on itself showing the substrate flexibility along with the semitransparency of the thin graphene film deposited on the surface. It is worth noting that, because they are held together solely by internanosheet van der Waals forces, binder-free nanosheet networks are mechanically very weak.^55^ As such, they are not particularly durable and can be easily removed from the substrate by abrasion. Thus, care must be taken when handling them. Any real application would certainly require encapsulation, perhaps via a sprayed polymer coating. A scanning electron microscope (SEM) image of the printed network, Figure 1F, shows a generally continuous network of nanosheets with some small pinholes. The Raman spectrum in Figure 1G shows the characteristic D, G and 2D graphene peaks, with the relatively low D peak intensity indicating that relatively few defects are present in the graphene, and the Lorentzian shape of the 2D peak is consistent with that expected for few-layer graphene.^53^

### Variation of Conductivity with Thickness

**Figure 2:**
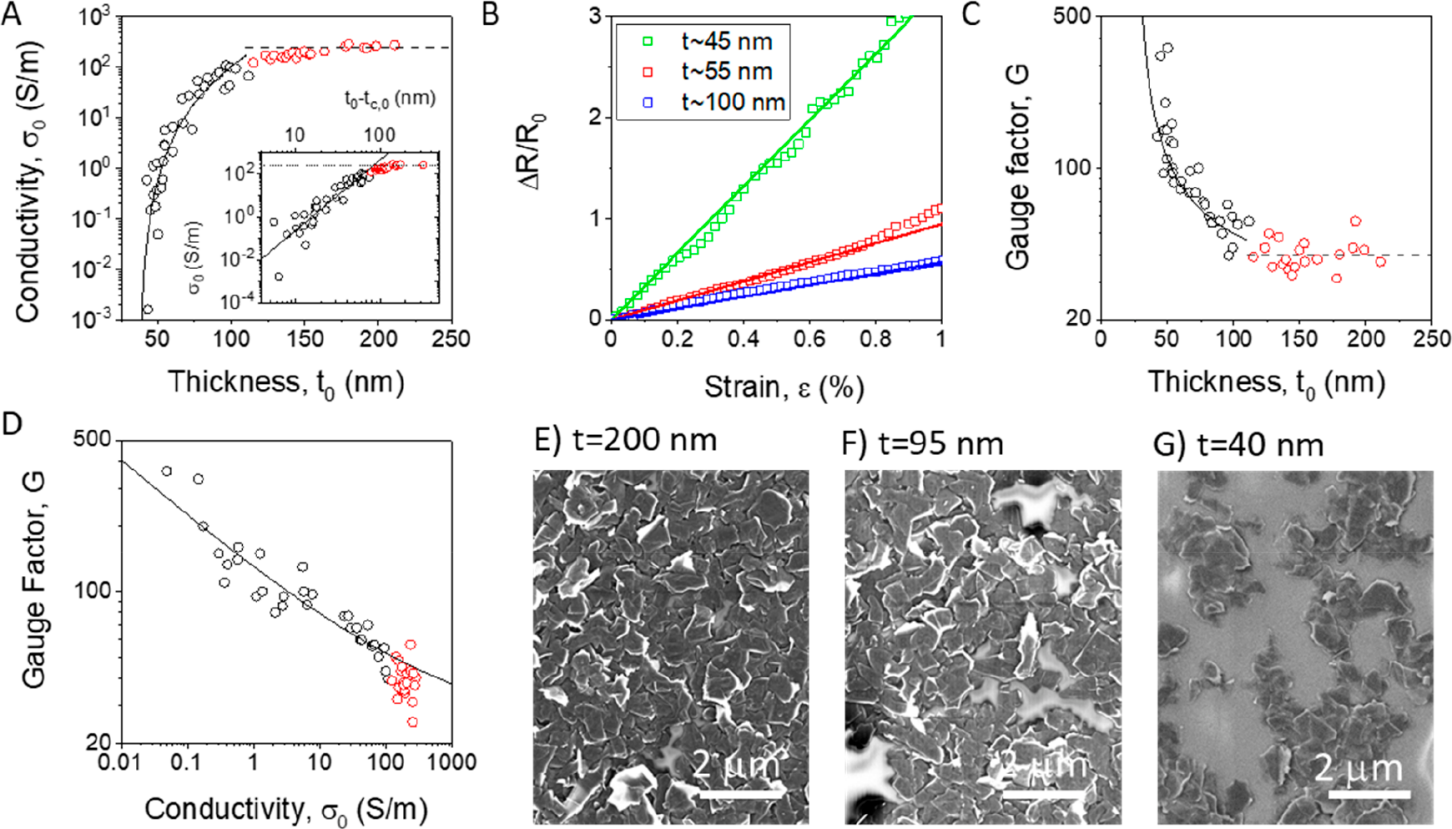
Printed nanosheet sensor characterization. (A) Plot of nanosheet film conductivity with thickness, measured at zero-strain (zero-strain data indicated by the subscript “0”). Inset log–log plot of σ_0_ versus (*t*_0_ – *t*_c,0_) with overlaid fit using eq 2a for *t*_0_ < 110 nm. The data for *t*_0_ > 110 nm are considered bulk-like and plotted in red with the average value indicated by the dashed line. (B) Fractional resistance change plotted versus strain for three representative sensors of varying film thickness. The solid lines are linear fits. (C) Gauge factor plotted against the thickness of the nanosheet film, with overlaid fit from eq 5 for *t*_0_ < 110 nm. The gauge factor plateaus for *t*_0_ > 110 nm in the bulk-like regime with an average of 39.5. (D) Plot of gauge factor as a function of conductivity, with overlaid fit from eq 6. Again, the data for *t*_0_ > 110 nm are considered bulk-like and plotted in red. (E–G) SEM images of three distinct sensors with thicknesses of 200, 95, and 40 nm, respectively. The decreasing surface coverage is evident as the thickness is decreased, and the few remaining nanosheet pathways can be seen in the 40 nm sample. Fit parameters for (A), (C), and (D) are given in Table 1.

We fabricated the piezoresistive sensors by using the dispersion described above as an ink, which was spray-coated onto highly stretchable PDMS substrates. This procedure resulted in semitransparent thin films (Figure 1E) consisting of disordered arrays of nanosheets (Figure 1F). We produced approximately 50 such films, varying the films thickness (measured by optical transmission, which was correlated to profilometry thickness (Supporting Information)) between ∼45 and 200 nm. For each film, we measured the electrical conductivity (in the absence of strain), σ_0_, which is plotted against film thickness (unstrained), *t*_0_, in Figure 2A. In all cases, the “0” subscript refers to zero-strain. This graph shows the conductivity increases with increasing thickness from ∼10^–3^ S/m for films of ∼45 nm thick before saturating above ∼150 nm at a conductivity of ∼260 S/m. We note that no measurable conductivity was found for networks thinner than 40 nm.

To understand this behavior, we note that conductivity is usually considered as an intrinsic material property, which is independent of the sample dimensions. However, this is not the case in thin, disordered, nanostructured films such as networks of graphene nanosheets or carbon nanotubes.^56^ While thick nanostructured films do indeed show thickness-independent, bulk-like conductivity, σ_B_, this is not the case for thin networks. Once the film thickness, *t*, falls below a critical value (*t*_x_), it has been observed that the conductivity decreases with decreasing film thickness. This effect is often referred to as percolation and is largely associated with disorder. The falloff in conductivity is linked with the reduction in number and connectivity of conductive pathways through the film, reducing its current carrying capacity. Eventually, for very thin films, a critical thickness, *t*_c_, is reached where only a single conductive pathway remains. This critical thickness (*t*_c_) is known as the percolation threshold, the minimum thickness where current will flow through the network.

Within this framework, the high-thickness, saturated conductivity observed in Figure 2A represents the bulk-like conductivity, σ_B_, while the thickness-varying conductivity at low film thickness represents the percolation regime. Such behavior has been observed in a number of systems including very thin networks of nanomaterials such as nanotubes, nanowires, and nanosheets and even thin evaporated metal films.^56,57^

Below *t*_x_, the thickness-dependent conductivity, σ, can be described quantitatively via percolation theory:^56,58^
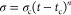
1where σ_c_ is a proportionality constant without physical meaning and with poorly defined units and *n* is the percolation exponent. However, as described above, when the network thickness exceeds a critical value (*t*_x_), then the conductivity saturates at a thickness-independent value, σ_B_, which can be associated with thick, bulk-like networks.^56^ At this critical thickness, σ_B_ = σ_c_(*t*_x_ – *t*_c_)^*n*^, allowing us to replace σ_c_ in eq 1, leading to
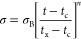
2a

This equation is superior to eq 1 as all parameters have clear physical meanings and well-defined units. Equation 2a is general and should apply even when strain is applied to the network, which means it can be used to analyze piezoresistive sensors. We expect the effect of strain will be to change the values of some or all of the parameters within the equation as compared to their unstrained values. In the absence of strain, each parameter simply takes on its zero-strain value, which we indicate via the subscript zero:
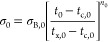
2b

Equation 2b has been fit to the data in Figure 2A for *t*_0_ < 110 nm (allowing for a transition region between thickness-dependent and thickness-independent regimes), as shown in the inset of Figure 2A. The fit is represented by the solid black line (reproduced in Figure 2A, main panel), which is consistent with *n*_0_ = 3.3 and *t*_c,0_ = 37 nm. In addition, for thick films, the data saturate at a constant value (dashed line) of σ_B,0_ = 260 S/m, while the crossover point of the solid and dashed lines yields *t*_x,0_ = 120 nm, values that are perfectly consistent with the fit. These parameters and their errors are summarized in Table 1.

**Table 1:** Fit Parameters Obtained from Fitting Data in Figure 2 to the Relevant Percolation Equations

parameter	value
From σ_0_ versus *t*_0_ (eq 2b)	
σ_B,0_	260 ± 20 S/m
*t*_c,0_	37 ± 5 nm
*t*_x,0_	120 ± 5 nm
*n*_0_	3.3 ± 0.3
From *G* versus *t*_0_ (eq 5)	
*G*_TNS_	22 ± 4
*t*_TNS_	2.3 ± 0.3 μm
*t*_c,0_	27 ± 3 nm
From *G* versus σ_0_ (eq 6)	
*G*_TNS_	21 ± 5
σ_TNS_	(3 ± 1) × 10^7^ S/m
*n*_0_	3.7 ± 0.3

### Electromechanical Properties

The literature would lead us to expect solution-processed nanosheet networks such as those above to display piezoresistive properties.^32,33,39,41^ However, it is not known whether, like the conductivity, the electromechanical response displays bulk-like and percolative regimes. To investigate this, the networks studied in Figure 2A were also subjected to electromechanical tests by straining from 0% to 1% strain at a rate of 1%/s with examples shown in Figure 2B. At low strain, the fractional resistance changes scales linearly with strain (ε) according to Δ*R*/*R*_0_ = *G*ε, allowing the networks to be used as strain sensors.^10^ Here, *G* is most properly considered as the slope of the Δ*R*/*R*_0_ versus ε curve at low strain. It is worth noting that these curves tend to be linear only up to ∼0.75–1% strain, which limits their utility to low-strain sensing. This is consistent with the literature where the linear response region for nanosheet-only strain sensors is typically below ∼5% strain.^41,42^ At higher strains, nonlinearities arise, with cracking of the network suggested as a major contributor.^59,60^ For comparison, we note that in composite systems the linear region generally extends well beyond 1% as shown comprehensively in a recent review.^12^ In that paper, linear regions as high as 100% strain were reported.^12,61^ A negative correlation between gauge factor and linear-strain-range was identified, suggesting that for systems where higher gauge factors are possible, the linear region only exists at low strain.

The gauge factor is plotted as a function of network thickness in Figure 2C. For thinner networks, *G* is highly thickness-dependent, behavior that has been alluded to in a small number of papers but not explored in detail.^33,41,48^ Interestingly, we observe a sharp increase in *G* for very low thickness leading to very high gauge factors of ∼350 for networks with thickness around 45 nm. Given that the percolation threshold is close to 40 nm, these data are consistent with a divergence in *G* as *t*_c_ is approached from above, behavior that is reminiscent of piezoresistive nanocomposites.^47^ Interestingly, similar to the conductivity data, *G* appears to be thickness-independent for thicknesses above about 120 nm. This behavior implies that, as with the conductivity data, the gauge factor displays both bulk-like and percolative regimes.

These low-thickness *G*-values compare favorably with literature reports for solution-processed graphene nanosheet films. Previous researchers have prepared strain sensors from graphene networks prepared by drop casting,^41^ inkjet printing,^32^ spray casting,^33^ and self-assembly,^39^ achieving gauge factors (at low-strain) of ∼10, 125, 170, and ∼300, respectively. Our best gauge factors (∼350) also compare favorably to polymer-based nanocomposite sensors. A 2019 study of 200 nanocomposite strain sensors^12^ ranked the reported *G*-values, which ranged from 0.01 to 2600.^22^ Our best sensors would rank fifth on this scale. While nanosheet networks and nanocomposite films are cheap and easy to prepare, more sophisticated methods have been used to make the highest sensitivity published sensors. For example, CVD grown films have yielded sensitivities as high as *G* = 300 [ref (26)] or even *G* = 600 for remote plasma-enhanced chemical vapor deposition (RPECVD) grown films.^42^

As mentioned above, for thicknesses greater than ∼120 nm, the gauge factor saturates with a mean value of 39 ± 1.6. The fact that both conductivity and gauge factor show thickness-independent behavior above *t*_x_ ≈ 120 nm but thickness-dependent behavior below this value suggests these parameters to be linked. To test this, we plot *G* versus σ_0_ in Figure 2D. We find a well-defined power-like decay, similar to that previously reported by Hu et al. for epoxy resin/carbon nanotube composites^62^ and by Garcia et al. for Sylgard/graphene composites.^47^ This relationship will be discussed in more detail below.

To better understand the nature of the significant increase in *G* as the thickness is reduced below *t*_0_ = *t*_x_ = 120 nm, we performed SEM analysis (Figure 2E–G) on networks of different thicknesses sprayed onto PDMS (note that *t*_x_ is the thickness where the electrical conductivity transitions from percolative to bulk-like). As the film thickness is reduced from 200 to 40 nm, the morphology of the networks changes drastically. Figure 2E shows a nanosheet network with *t* = 200 nm. This is in the bulk-like conductivity regime and is continuous with very few holes. Shown in Figure 2F is a *t* = 95 nm network, which is just below *t*_x_ = 120 nm. Here, the network is less uniform, with the PDMS substrate visible through numerous gaps in network. The SEM image in Figure 2G is of a *t* = 40 nm thick sprayed film, which is very close to the percolation threshold, *t*_c_. Here, the network is extremely nonuniform with the PDMS substrate clearly visible and individual current carrying pathways easily identifiable. These nonuniformities are responsible for the percolating conductivity below *t*_x_ and probably play a role in the increased gauge factor in this regime. For highly nonuniform networks, the current carrying capacity of the film is now dependent on fewer current paths. This means that the strain-induced disruption of a few nanosheet junctions can have a significant impact on network resistance.

### Modeling the Piezoresistance of Thin Networks

This observed dependence of *G* on both network thickness and conductivity is reminiscent of nanocomposite strain sensors where similar behavior is observed (although there, σ_0_ and *G* scale with the filler volume fraction, rather than the film thickness). Recently, we were able to quantitatively explain such behavior in composites using a simple model.^47^ When a material is strained, the resistance changes partly because of a relatively small change in sample dimensions, but more importantly due to variations in the material conductivity with strain.^10^ The second effect can be positive^11,63^ or negative^46^ and can be very large in some systems,^11^ especially nanocomposites. It is well-known that considering both effects leads to a simple equation [see refs (11), (19), and the Supporting Information]:
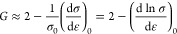
3where the subscript zero means the quantity must be taken at low-strain such that σ_0_ denotes the zero-strain conductivity. This low-strain condition comes from approximations in the derivation that are valid only at low-strain (see the Supporting Information).

Following our previous approach, we can apply this equation to a nanosheet (or any other nanoparticle) network by differentiating eq 2a with respect to strain, assuming σ_B_, *n*, *t*_x_, *t*_c_, and *t* all depend on strain. Performing this differentiation (see the Supporting Information) yields an expression for *G* in terms of all five parameters in eq 2a and their zero-strain derivatives:
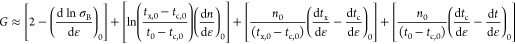
4a

Although it looks complicated, this equation is actually quite simple and shows how the gauge factor *G* should depend on the film thickness at zero-strain, *t*_0_. In fact, it is quite similar to the equivalent equation for piezoresistive nanocomposites,^47^ although the third, square-bracketed term does not exist in nanocomposites.

In addition, (d*t*/dε)_0_ does not appear explicitly in the nanocomposite model^47^ (although it is included implicitly). Defining the relevant Poisson ratio as the ratio of strain in the film transverse (thickness) direction (ε_t_) to that in the longitudinal (in-plane) direction (ε), *v*_tL_ = −dε_t_/dε, it is straightforward to show that (d*t*/dε)_0_ = −*v*_tL_*t*_0_. For highly porous, nanostructured systems, the Poisson ratio can be very small (often −0.1 < *v*_tL_ < 0.1).^64−66^ We argue that this allows us to neglect the (d*t*/dε)_0_ term, although this approximation should be made on a case by case basis and properly justified (as we do below). This analysis can also be applied to the third term: (d(*t*_x_ – *t*_c_)/dε)_0_ = −*v*_tL_(*t*_x,0_ – *t*_c,0_). This means the third term is equal to −*v*_tL_*n*_0_, which can be neglected if we assume *v*_tL_ is small. N.B. This process cannot be used to eliminate (d*t*_c_/dε)_0_ in the fourth term in eq 4a as it is clear from the experimental data that this term is dominant, especially for thin networks, and cannot be neglected.

Combining these approximations, eq 4a becomes
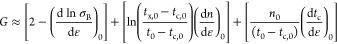
4bwhich is a reasonably simple yet physically descriptive representation of the piezoresistive response in nanosheet networks. We note that the physical significance of the three square-bracketed terms is defined by the physical significance of the percolation parameters (σ_B_, *n*, and *t*_c_), whose strain-derivatives are contained in each. The physical significance of these parameters has been discussed elsewhere.^47^ In brief, dσ_B_/dε (and so the first term) is controlled by the effect of strain on internanosheet charge transport; d*n*/dε is determined by the effect of strain on network structure and dimensionality, while d*t*_c_/dε is determined by the effect of strain on the network structure.^11,47,67^

### Simple Equations for Data Fitting

Even in its simplified form, eq 4b has too many parameters for effective data fitting. However, a further simplification can be achieved by noting that, although the second, square-bracketed term depends on *t*_0_, the dependence is weak as compared to the final term (see ref (47)). This allows us to approximate the first two terms as thickness-independent, writing their sum as *G*_TNS_, where TNS stands for “thin network sensor”.We then can write eqs 4a and 4b as
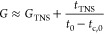
5where *t*_TNS_ is a constant (units: m) given by *t*_TNS_ ≈ *n*_0_(d*t*_c_/dε)_0_. Both *G*_TNS_ and *t*_TNS_ are figures-of-merit for thin network sensors with larger values of both parameters leading to higher sensor sensitivity.

As shown in Figure 2C, we have fit the *G* versus *t*_0_ data using eq 5. We have limited the fit to values of *t*_0_ less than 110 nm, consistent with the region where the electrical percolation data (Figure 2A) were fitted. We find a good fit with values of *G*_TNS_ = 22 ± 4, *t*_TNS_ = 2.3 ± 0.3 μm, and *t*_c,0_ = 27 ± 3 nm (Table 1). We note that *t*_c,0_ is similar but not identical to that found from the electrical percolation fit. Combining this value of *t*_TNS_ with the value of *n*_0_ = 3.3 obtained from the electrical percolation fitting, and assuming (d*t*_c_/dε)_0_ ≫ |*v*_tL_*t*_0_| as described above, allows us to estimate (d*t*_c_/dε)_0_ = 700 nm, which is equivalent to an increase in *t*_c_ by 7 nm for every percentage of applied strain. Given that the maximum value of *t*_0_ in the percolative regime is ∼120 nm and the Poisson ratio cannot be greater than 0.5,^68^*v*_tL_*t*_0_ has a maximum value of 60 nm, validating our initial assumption.

From a physics standpoint, eq 5 sheds light on what factors most strongly influence the piezoresistive response. For thin networks (*t*_0_ ≪ *t*_x_) with large *G*-values, the second term in eq 5 completely dominates the gauge factor. The magnitude of this term is largely set by *t*_TNS_, which is in turn sensitive to (d*t*_c_/dε)_0_ (*n*_0_ is usually quite close to 2 for such networks^69^). Because (d*t*_c_/dε)_0_ is a measure of the sensitivity of the percolation threshold to strain, and hence is a measure of the impact of strain on the structure of the network, this means the second term in eq 5 is associated with the network morphology rather than the effect of strain on interparticle junctions as is usually thought (this effect is contained in the first term in eqs 4a and 4b and so the first term in eq 5).

We can also combine eq 5 with eq 2b to express *G* as a function of the zero-strain conductivity of the network (σ_0_):
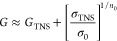
6where σ_TNS_ = σ_B,0_(*t*_TNS_/(*t*_x,0_ – *t*_c,0_))^*n*_0_^ is a constant for which large values are associated with higher *G*. This equation can be used to fit the *G* versus σ_0_ data plotted in Figure 2D (for *t*_0_ < 110 nm). Fitting yields *G*_TNS_ = 22 ± 4, σ_TNS_ = (3 ± 1) × 10^7^ S/m, and *n*_0_ = 3.7 ± 0.3 (Table 1). Clearly, the values of *G*_TNS_ and *n*_0_ are very similar to those quoted above. The utility of eq 6 is that it predicts and explains the well-defined power-law relationship between *G* and conductivity that has been alluded to by previous authors.^47^

### Contribution of Intrananosheet versus Internanosheet Charge Transport to *G*

The first term in eq 4b contains information about the strain dependence of σ_B_, the conductivity of a bulk-like nanosheet network. It has been argued previously that the conductivity of a nanosheet network scales inversely with *R*_NS_ + *R*_J_, the sum of the resistances of an individual nanosheet and an individual junction.^11,67^ As shown in the Supporting Information, this allows us to write
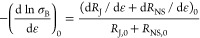
7awhere, as usual, the subscript zeros indicate zero-strain. If we define gauge factors associated with the nanosheet itself and the internanosheet junction as (d*R*_NS_/dε)_0_ = *G*_NS_*R*_NS,0_ and (d*R*_J_/dε)_0_ = *G*_J_*R*_J,0_, then eq 7a can be rearranged as
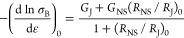
7b

This equation allows us to separate the contributions of the intrinsic piezoresistive mechanism associated with the graphene nanosheet from that of the internanosheet junction. It has recently been shown that for networks of graphene nanosheets (as well as other conducting 2D nanosheets), the ratio (*R*_NS_/*R*_J_)_0_ ≪ 1.^67^ In addition, once extrinsic factors such as cracking or intergrain tunneling are absent, it is known that *G*_NS_ is quite small, <10 for graphene sheets.^35−37^ This means we expect the contribution of nanosheet piezoresistance to the network piezoresistance to be very small. Applying the approximations above allows us to write
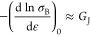
7c

This shows that the first term in eq 4b is dominated by the effect of strain on internanosheet junctions. In fact, it is widely believed that this phenomenon dominates the piezoresistance of the conducting networks.^62,70−72^

However, it must be emphasized that the fit in Figure 2C shows that the first term in eq 5 and so the first two terms in eq 4b (i.e., those terms related to *G*_J_) only make a significant contribution to *G* for thick networks. For thinner networks, *G* is dominated by the last term in these equations, which is controlled by (d*t*_c_/dε)_0_, and so network structure.^47^ This means that those networks with the highest gauge factors are not predominantly limited by the effect of strain on internanosheet junctions as is commonly believed.

Incidentally, because the first term in eq 4b is the only one that applies to bulk-like films, this means that eq 7c coupled with eq 4b determines the gauge factor of thick films: *G*_bulklike_ = 2 + *G*_J_. Combined with the data for thicknesses greater than ∼120 nm, this means that *G*_J_ = 37 ± 1.6.

### Cyclability, Hysteresis, and Frequency Dependence

Academic literature on strain sensors usually focuses on the gauge factor. However, as mentioned in the Introduction, other factors are also important. These include low hysteresis, minimal frequency dependence of *G*, and good cyclability. Here, we will investigate these.

**Figure 3:**
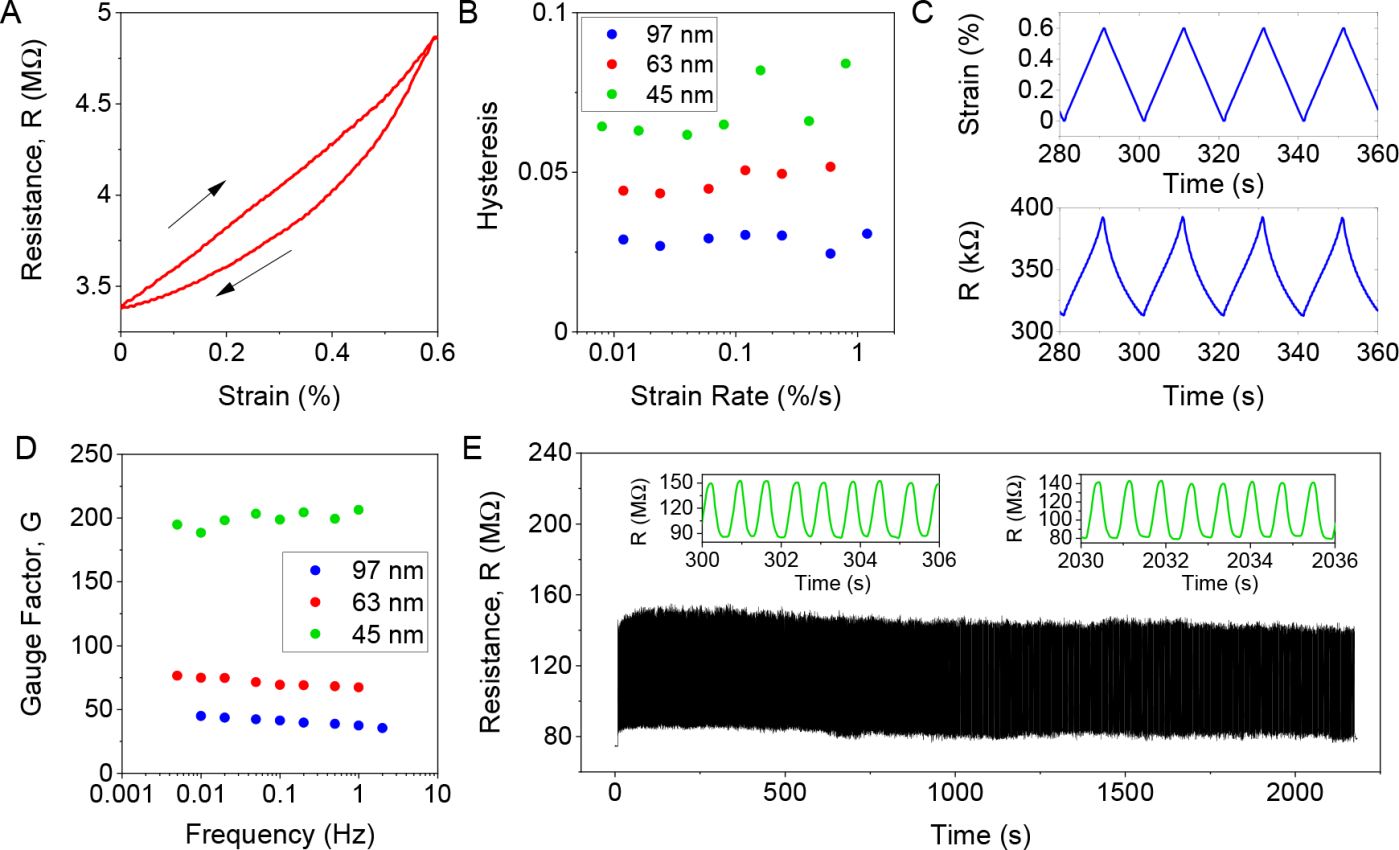
Hysteresis and cyclability testing. (A) Resistance hysteresis profile as a function of strain for the *t*_0_ = 63 nm film, measured with a strain rate of 0.024%/s. (B) Comparison of hysteresis as a function of strain rate for three sensors of different thicknesses, showing the reasonable stability of hysteresis across two decades of strain rate and the increase of hysteresis at lower thicknesses (max strain used was 0.6% except for the 45 nm sample where it was reduced to 0.4% to avoid damage after repeated cycling). (C) Cyclic resistance response of 97 nm thick sensor with 0.05 Hz sawtooth cycling profile as shown. (D) Comparison of gauge factor as a function of cyclic frequency for three sensors, showing near frequency independence from 0.01 to 1 Hz. Note that the strain amplitudes were 0.4% for the 45 nm thick film and 0.6% for the 63 and 97 nm films. (E) Cyclic testing (sawtooth ∼1.5 Hz), 0–0.4% strain) for the *t*_0_ = 45 nm film showing stability over 3000 cycles. The inset shows the magnified regions at the start and end of the cycling profile.

Hysteresis is present when the resistance–strain curve during unloading does not follow the initial path traced out during loading and implies that the loading process has (at least temporarily) altered the structure of the network.^14,15^ We note that, to the authors’ knowledge, there are no detailed explanations of the origin of hysteresis in the literature. We define the hysteresis of a strain sensor as the area within the hysteresis loop in a resistance versus strain plot as the sensor is loaded and released, divided by the area under the resistance versus strain curve for loading. An example of a hysteresis loop is shown in Figure 3A for a *t*_0_ = 63 nm sensor deformed at a strain rate of 0.024%/s to a maximum strain of 0.6% before unloading. In this case, the hysteresis value was 5%. As shown in Figure 3B, the hysteresis is roughly constant across 2 orders of magnitude of strain rate for three different film thicknesses, all of which show less than 10% hysteresis. Interestingly, the thinner films appear to have higher hysteresis with a well-defined inverse relationship observed empirically. It is very difficult to put the hysteresis values in context as, although some papers measure hysteresis,^74,75^ very few quote a numerical value. However, we can say that these results compare favorably to printed polymer–graphene composites, which demonstrated a hysteresis of ∼15% [ref (13)].

For real applications, strain sensors must be able to monitor cyclic strains at multiple frequencies. Under these circumstances, it is imperative that *G* is frequency-invariant across a range of frequencies and over thousands of straining cycles. To test this, we applied a sawtooth 0.05 Hz cyclic strain profile to a *t*_0_ = 97 nm sensor (Figure 3C, top). The corresponding resistance shows the high stability of the gauge factor from cycle to cycle. Figure 3D shows the resultant dynamic gauge factor plotted versus frequency (all sawtooth profiles), for three different network thicknesses. There is good stability in the gauge factor across over 2 orders of magnitude of frequency in all three sensors. The thinnest films show a high dynamic gauge factor of *G* ≈ 200. Although very few papers report frequency-dependent piezoresistive results, our results are consistent with those of Qiao et al. and Li et al., which both report frequency-invariant behavior.^39,40^Figure 3E demonstrates the stability of the 45 nm thick film over 3000 cycles. The inset plots show zoomed-in profiles at the start and end of the 3000 cycles showing good fidelity and consistent gauge factors of *G* ≈ 187 and *G* ≈ 184, respectively.

**Figure 4:**
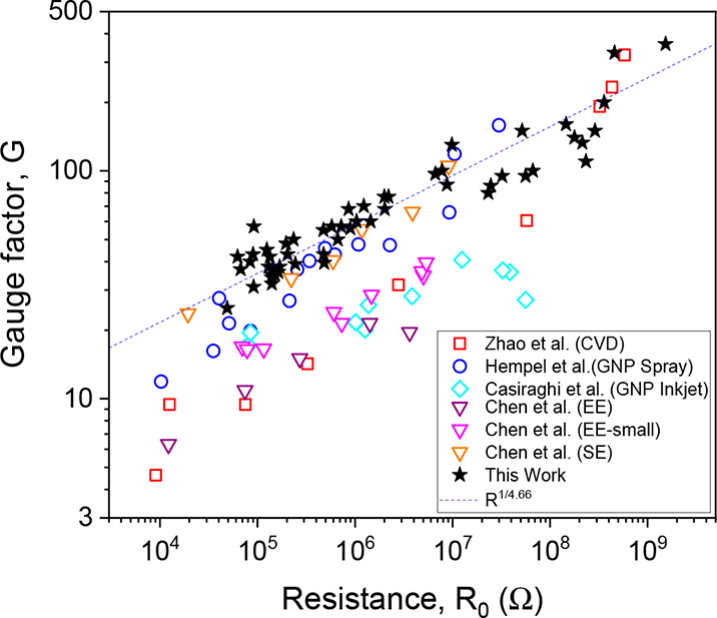
Comparison of our results with previous literature plotted as gauge factor versus zero-strain sensor resistance. Papers used in the analysis: Zhao et al. (CVD),^26^ Hempel et al. (spray),^33^ Casiraghi et al. (inkjet on paper),^32^ and Chen et al. (electrochemical exfoliation/EE-small (sonicated after exfoliation)/solvent exfoliated).^78^ The dashed line shows power law dependence with an exponent of 1/(*n*_0_ + 1) = 1/4.66.

Finally, to put our results in context, we compare our gauge factor data with literature data for graphene-only strain sensors prepared by both solution processing as well as CVD (Figure 4). To do this, we plot the gauge factor versus the sensor resistance (at zero strain). Plotting versus resistance rather than conductivity is necessary as most papers do not quote sensor thickness, making calculation of conductivity impossible. The most obvious feature of this graph is that all data sets show a roughly power law correlation between gauge factor and resistance. To explain this, we note that, according to eq 2b, so long as the network is well above the percolation threshold (*t*_0_ ≫ *t*_c,0_), then σ_0_ ∝ *t*_0_^*n*_0_^, which means that the zero-strain resistance scales with (unstrained) film thickness as *R*_0_ ∝ *t*_0_^–(*n*_0_ + 1)^. Applying eq 5 means that *G* – *G*_TNS_ ∝ *R*_0_^1/(*n*_0_ + 1)^ (when *t*_0_ ≫ *t*_c,0_). Assuming both *t*_c,0_ and *G*_TNS_ are relatively small, this predicts the observed power law relationship *G* ∝ *R*_0_^1/(*n*_0_ + 1)^. To confirm this, we plot the dashed line, which has an exponent of 1/4.66, consistent with the percolation exponent of 3.66. Perhaps usefully, this relationship allows the percolation exponent to be extracted from the measurements on a set of films of unknown thickness. In addition, it is worth pointing out that the gauge factors reported here are competitive with the best graphene-based gauge factors reported in the literature, even for CVD-based sensors.

Because of their excellent performance and ease of fabrication, we believe printed graphene networks have significant potential for use as practical strain sensors. However, much engineering work is required to move these structures from promising sensing materials to practical components within sensors. For example, as indicated above, methods will have to be developed to encapsulate the networks without significant reduction in either conductivity or gauge factor. In addition, it will be important to quantify the effects of humidity on the network properties^76^ and assess whether any negative impact can also be ameliorated by encapsulation. Moreover, it is well-known that nanonetworks can have gauge factors that have nontrivial temperature dependences.^77^ It will be important to assess the temperature dependence of *G* and identify a regime where the temperature variation is minimized. In this particular area, our results may be useful. Any dependence of *G* on temperature is likely to stem from the first term in eqs 4a and 4b as this term is linked to internanosheet hopping, which is temperature-dependent. However, this work shows the relative influence of this term to be minimized as the network thickness is reduced toward *t*_c,0_. Thus, thickness control may be a strategy to minimize the temperature variation of the gauge factor.

## Conclusion

We have performed a detailed study on the dependence of both electrical conductivity and piezoresistive properties, notably gauge factor, on the thickness of printed networks of graphene nanosheets. We find that both conductivity and gauge factor are thickness-independent (i.e., intrinsic) properties for networks thicker than ∼120 nm. However, both conductivity and gauge factor depend sensitively on nanosheet thickness for thinner networks. We show that the thickness-dependence of gauge factor is closely related to that of conductivity and that both can be quantitatively described by percolation theory. In addition, we find these sensors to have low-hysteresis and good frequency independence and to demonstrate excellent cyclability over thousands of cycles.

## Experimental Methods

### Ink Preparation

Graphene ink was prepared using liquid-phase exfoliation (LPE) of graphite flakes (Branwell, graphite grade RFL 99.5, 20 g) in 1-methyl-2-pyrrolidone (Sigma-Aldrich, 200 mL) by tip sonication (200 W, 70% amplitude, 72 h, Hielscher UP200S, 200 W, 24 kHz). This dispersion underwent centrifugation (Hettich Mikro 220R) at 1500 rpm (RCF = 230*g*) for 90 min to remove large nanosheets and unexfoliated bulk graphite. The supernatant was vacuum filtered through a 0.45 μm nylon membrane (Sterlitech NY4547100), forming a pellet of graphene. This was washed through by adding methanol (Sigma-Aldrich, 30 mL). The residual membrane was dried in a vacuum oven (Fi-Streem Vacuum Oven) at 50 °C overnight. The carbon membrane was weighed (Sartorius Balance), ground into a powder using a mortar and pestle, and resuspended in chloroform (Sigma-Aldrich) by tip sonication for 1 h at 40% amplitude to make a graphene/chloroform dispersion with a concentration of 2 mg/mL. The dispersion concentration and nanosheet thickness were estimated using UV–vis characterization (Cary 50) as outlined by Backes et al.^53^ This dispersion was diluted to the required concentrations for spray printing.

### Substrate Preparation

PDMS Sylgard 184 Dow Corning substrates were fabricated by mixing components A (2.00 mL, silicone oil base) and B (200 μL, curing agent) in a 10:1 volume ratio in a PTFE mold. These were cured in an oven (2 h, 120 °C), after which the cured PDMS was removed from the mold and cut into strips of the required size.

### Spraying

Thin films of graphene were deposited by spray coating from a modified airbrush (Harder & Steenbeck Infinity Airbrush), which was mounted in a mobile gantry (Janome JR2300N). The gantry was programmed to raster across a 5 cm × 5 cm area where the substrates were held in place using Kapton tape. The working distance from the nozzle to substrates was 10 cm, and the nitrogen back pressure was set to 3.5 bar. Films with thickness above 100 nm were reasonably uniform with well-defined thicknesses that could be measured by a profilometer. Thinner films tend to display more inhomogeneous morphologies. However, the average thicknesses (see below) measured for such thin films were reasonably repeatable. For example, a batch of six sprayed films typically displayed a thickness variation of <15 nm.

### Thickness Characterization

Graphene film thickness was characterized using a flatbed scanner (Epson Perfection V700 PHOTO) to determine the optical transmission and so extinction. The scanner was calibrated using neutral density filters of known transmission. The film extinction was converted to film thickness using the extinction coefficient, which was measured using sprayed films of the same graphene dispersion on glass, whose thickness was measured using profilometry. The extinction coefficient was measured for thicker films using between 100 and 250 nm for which the profilometry was more reliable. Very thin films can be somewhat inhomogeneous, making the thickness poorly defined. Measuring the thickness from the optical transmission, as we do here, then is equivalent to measuring an average thickness.

### Electromechanical Testing

Sensors were tested using a Zwick Z0.5 ProLine Tensile Tester (100 N Load Cell). The films were contacted using silver wires attached using silver paint directly on the graphene film. Sample dimensions were approximately 5 mm × 25 mm with PDMS thickness in the range of 0.7–1.0 mm. Sensors were conditioned by sawtooth profile strain cycling before testing. Conditioning is particularly important as otherwise the initial stretch/release cycle can give an unrepresentative, anomalous electrical response. It is likely that the as-produced network is in a nonequilibrium state and conditioning leads to a slight reorganization of the network into a more stable state. This final state likely has improved connectivity as the total resistance tends to decrease over the conditioning cycle. The resistance was measured using a Keithley KE2601 Source meter controlled by a 2-probe LabView program. Electromechanical measurements were made using a maximum strain amplitude of 1%. Straining beyond 1% tended to lead to irreversible cracking or delamination of electrodes. Cyclic measurements were initially performed at 0.6% strain amplitude. However, we found that 0.6% strain eventually led to damage to the 45 nm film after repeated cycling. Subsequently, all long cycling experiments on the 45 nm sample were performed with a 0.4% strain amplitude, which could be applied for many cycles without damage appearing.

## Supporting Information Available

Detailed derivations, network characterization, and literature comparison (PDF)
