# Supplementary material for: Quantifying the Piezoresistive Mechanism in High-Performance Printed Graphene Strain Sensors
Source: ACS Appl Mater Interfaces. 2022 Jan 31;14(5):7141–51. doi: 10.1021/acsami.1c21623 (PMC8832394; doi:10.1021/acsami.1c21623)
Supplement: Supplementary file 1 — am1c21623_si_001.pdf [file am1c21623_si_001.pdf]

Supporting Information:

Quantifying the Piezoresistive Mechanism in High Performance Printed Graphene Strain Sensors

Eoin Caffrey, James R Garcia, Domhnall O'Suilleabhain, Cian Gabbett, Tian Carey and Jonathan N. Coleman[1]*

[1]*School of Physics, CRANN & AMBER Research Centres, Trinity College Dublin, Dublin 2, Ireland*

*colemaj@tcd.ie (Jonathan N. Coleman); Tel: +353 (0) 1 8963859.

Gauge Factor Derivation

The Gauge Factor (G) is typically defined in terms or resistance (R) and strain (ε), where subscript zero indicates at zero strain.

$$\frac{\Delta R}{R_0} = G\varepsilon \tag{S.1}$$

Hence, at low strain $G = \frac{1}{R_0}\frac{dR}{d\varepsilon}$. Resistance can be expressed in terms of the length (L), cross sectional area (A) and the conductivity (σ).

$$R = \frac{L}{\sigma A} \tag{S.2}$$

At low strain, assume the volume remains constant.

$$AL = A_0 L_0 \tag{S.3}$$

$$R = \frac{L^2}{\sigma A_0 L_0} \tag{S.4}$$

Differentiating with respect to strain

$$\frac{dR}{d\varepsilon} = \frac{1}{A_0 L_0}\left[\frac{2L}{\sigma}\frac{dL}{d\varepsilon} - \frac{L^2}{\sigma^2}\frac{d\sigma}{d\varepsilon}\right] \tag{S.5}$$

Dividing through by $R_0 = \frac{L_0}{\sigma_0 A_0}$

$$\frac{1}{R_0}\frac{dR}{d\varepsilon} = \frac{\sigma_0}{L_0^{\ 2}}\left[\frac{2L}{\sigma}\frac{dL}{d\varepsilon} - \frac{L^2}{\sigma^2}\frac{d\sigma}{d\varepsilon}\right] \tag{S.6}$$

Taking the limit of low strain, $L \approx L_0$ and $\sigma \approx \sigma_0$.

$$\frac{1}{R_0}\frac{dR}{d\varepsilon} \approx \frac{\sigma_0}{L_0^{\ 2}}\left[\frac{2L_0}{\sigma_0}\frac{dL}{d\varepsilon} - \frac{L_0^{\ 2}}{\sigma_0^{\ 2}}\frac{d\sigma}{d\varepsilon}\right] = \frac{2}{L_0}\frac{dL}{d\varepsilon} - \frac{1}{\sigma_0}\frac{d\sigma}{d\varepsilon} \tag{S.7}$$

As strain is defined as $\varepsilon = \frac{L - L_0}{L_0}$ we can rewrite the above expression as

$$G = \frac{1}{R_0}\frac{dR}{d\varepsilon} \approx 2 - \frac{1}{\sigma_0}\left(\frac{d\sigma}{d\varepsilon}\right)_0 \tag{S.8}$$

Gauge Factor Model Derivation

The percolation dependence of thickness on conductivity (σ) can be expressed in terms of thickness (t), percolation thickness (t_c), the percolation exponent (n) and a proportionality constant (σ_c).

$$\sigma = \sigma_c (t - t_c)^n \tag{S.9}$$

Conductivity can saturate above a critical thickness (t_x) and so a more complete form of the expression is given below, where σ_{Bulk} is the bulk conductivity of the network above t_x.

$$\sigma = \sigma_{Bulk} \left(\frac{t - t_c}{t_x - t_c} \right)^n \tag{S.10}$$

From Eq.S.8 we know that

$$G \simeq 2 - \frac{1}{\sigma_0} \left(\frac{d\sigma}{d\varepsilon} \right)_0 \tag{S.11}$$

Differentiating Eq.S.10 with respect to strain gives

$$\frac{d\sigma}{d\varepsilon} = \frac{\partial\sigma}{\partial\varepsilon} + \frac{\partial\sigma}{\partial\sigma_{Bulk}} \frac{d\sigma_{Bulk}}{d\varepsilon} + \frac{\partial\sigma}{\partial n} \frac{dn}{d\varepsilon} + \frac{\partial\sigma}{\partial t_x} \frac{dt_x}{d\varepsilon} + \frac{\partial\sigma}{\partial t} \frac{dt}{d\varepsilon} + \frac{\partial\sigma}{\partial t_c} \frac{dt_c}{d\varepsilon} \tag{S.12}$$

$$\frac{\partial\sigma}{\partial\varepsilon} = 0 \tag{S.13}$$

$$\frac{\partial\sigma}{\partial\sigma_{Bulk}} = \left(\frac{t - t_c}{t_x - t_c} \right)^n \tag{S.14}$$

$$\frac{\partial\sigma}{\partial n} = \ln\left(\frac{t - t_c}{t_x - t_c} \right) \left(\sigma_{Bulk} \left(\frac{t - t_c}{t_x - t_c} \right)^n \right) \tag{S.15}$$

$$\frac{\partial\sigma}{\partial t_x} = -\frac{n}{t_x - t_c} \left(\sigma_{Bulk} \left(\frac{t - t_c}{t_x - t_c} \right)^n \right) \tag{S.16}$$

$$\frac{\partial\sigma}{\partial t} = \frac{n}{t - t_c} \left(\sigma_{Bulk} \left(\frac{t - t_c}{t_x - t_c} \right)^n \right) \tag{S.17}$$

$$\frac{\partial \sigma}{\partial t_c} = \left(\frac{n}{t_x - t_c} - \frac{n}{t - t_c} \right) \left(\sigma_{Bulk} \left(\frac{t - t_c}{t_x - t_c} \right)^n \right) \quad \text{(S.18)}$$

Substituting each of these derivatives into Eq.S.12 gives the below expression

$$\frac{d\sigma}{d\varepsilon} = \left(\frac{t - t_c}{t_x - t_c} \right)^n \frac{d\sigma_{Bulk}}{d\varepsilon} + \ln\left(\frac{t - t_c}{t_x - t_c} \right) \left(\sigma_{Bulk} \left(\frac{t - t_c}{t_x - t_c} \right)^n \right) \frac{dn}{d\varepsilon} - \frac{n}{t_x - t_c} \left(\sigma_{Bulk} \left(\frac{t - t_c}{t_x - t_c} \right)^n \right) \frac{dt_x}{d\varepsilon}$$
$$+ \frac{n}{t - t_c} \left(\sigma_{Bulk} \left(\frac{t - t_c}{t_x - t_c} \right)^n \right) \frac{dt}{d\varepsilon} + \left(\frac{n}{t_x - t_c} - \frac{n}{t - t_c} \right) \left(\sigma_{Bulk} \left(\frac{t - t_c}{t_x - t_c} \right)^n \right) \frac{dt_c}{d\varepsilon}$$

(S.19)

This is simplified by dividing through by $\sigma = \sigma_{Bulk} \left(\frac{t - t_c}{t_x - t_c} \right)^n$

$$\frac{1}{\sigma}\frac{d\sigma}{d\varepsilon} = \frac{1}{\sigma_{Bulk}}\frac{d\sigma_{Bulk}}{d\varepsilon} + \ln\left(\frac{t - t_c}{t_x - t_c} \right) \frac{dn}{d\varepsilon} - \frac{n}{t_x - t_c}\frac{dt_x}{d\varepsilon} + \frac{n}{t - t_c}\frac{dt}{d\varepsilon} + \left(\frac{n}{t_x - t_c} - \frac{n}{t - t_c} \right) \frac{dt_c}{d\varepsilon}$$

(S.20)

As the gauge factor is only defined in the limit of low strain, take all derivatives in the limit of low strain and let $\sigma \simeq \sigma_0$ and as a result $\sigma_{Bulk} \simeq \sigma_{Bulk,0}, n \simeq n_0$, $t_x \simeq t_{x,0}$, $t \simeq t_0$ and $t_c \simeq t_{c,0}$

$$\frac{1}{\sigma_0}\left(\frac{d\sigma}{d\varepsilon} \right)_0 = \frac{1}{\sigma_{Bulk,0}}\left(\frac{d\sigma_{Bulk}}{d\varepsilon} \right)_0 + \ln\left(\frac{t_0 - t_{c,0}}{t_{x,0} - t_{c,0}} \right) \left(\frac{dn}{d\varepsilon} \right)_0 - \frac{n_0}{t_{x,0} - t_{c,0}} \left(\frac{dt_x}{d\varepsilon} \right)_0$$
$$+ \frac{n_0}{t_0 - t_{c,0}} \left(\frac{dt}{d\varepsilon} \right)_0 + \left(\frac{n_0}{t_{x,0} - t_{c,0}} - \frac{n_0}{t_0 - t_{c,0}} \right) \left(\frac{dt_c}{d\varepsilon} \right)_0 \quad \text{(S.21)}$$

Substituting Eq.S.21 into Eq.S.11 yields

$$G \simeq 2 - \frac{1}{\sigma_{Bulk,0}}\left(\frac{d\sigma_{Bulk}}{d\varepsilon} \right)_0 - \ln\left(\frac{t_0 - t_{c,0}}{t_{x,0} - t_{c,0}} \right) \left(\frac{dn}{d\varepsilon} \right)_0 + \frac{n_0}{t_{x,0} - t_{c,0}} \left(\frac{dt_x}{d\varepsilon} \right)_0 - \frac{n_0}{t_0 - t_{c,0}} \left(\frac{dt}{d\varepsilon} \right)_0$$
$$- \left(\frac{n_0}{t_{x,0} - t_{c,0}} - \frac{n_0}{t_0 - t_{c,0}} \right) \left(\frac{dt_c}{d\varepsilon} \right)_0 \quad \text{(S.22)}$$

Applying the relation in Eq.S.23 and by grouping the like terms, G is shown to depend on four terms, each in square brackets.

$$\frac{1}{\sigma_{Bulk}}\frac{d\sigma_{Bulk}}{d\varepsilon} \simeq \frac{d\ln(\sigma_{Bulk})}{d\varepsilon} \tag{S.23}$$

$$G \simeq \left[2-\left(\frac{d\ln(\sigma_{Bulk})}{d\varepsilon}\right)_0\right]+\left[\ln\left(\frac{t_{x,0}-t_{c,0}}{t_0-t_{c,0}}\right)\left(\frac{dn}{d\varepsilon}\right)_0\right]+\left[\left(\frac{n_0}{t_{x,0}-t_{c,0}}\right)\left\{\left(\frac{dt_x}{d\varepsilon}\right)_0-\left(\frac{dt_c}{d\varepsilon}\right)_0\right\}\right]$$
$$+\left[\frac{n_0}{t_0-t_{c,0}}\left\{\left(\frac{dt_c}{d\varepsilon}\right)_0-\left(\frac{dt}{d\varepsilon}\right)_0\right\}\right]$$

(S.24)

The first three terms are approximated as thickness independent, hence, we can write these in a simplified form as G_{TNS}. It is noted that the second term does have some thickness dependence, however, as discussed in the main text this dependence is weak compared to the final term.

$$G_{TNS} \simeq \left[2-\left(\frac{d\ln(\sigma_{Bulk})}{d\varepsilon}\right)_0\right]+\left[\ln\left(\frac{t_{x,0}-t_{c,0}}{t_0-t_{c,0}}\right)\left(\frac{dn}{d\varepsilon}\right)_0\right]+\left[\left(\frac{n_0}{t_{x,0}-t_{c,0}}\right)\left\{\left(\frac{dt_x}{d\varepsilon}\right)_0-\left(\frac{dt_c}{d\varepsilon}\right)_0\right\}\right]$$

(S.25)

Furthermore, it is noted that the third term in Eq.S.24 can be omitted in this case, by assuming that v_{tL} is small, as in the main text. Therefore, G_{TNS} is approximated as

$$G_{TNS} \simeq \left[2-\left(\frac{d\ln(\sigma_{Bulk})}{d\varepsilon}\right)_0\right]+\left[\ln\left(\frac{t_{x,0}-t_{c,0}}{t_0-t_{c,0}}\right)\left(\frac{dn}{d\varepsilon}\right)_0\right] \tag{S.26}$$

The final term of Eq.S.24 contains the following difference of derivatives, $\left\{\left(\frac{dt_c}{d\varepsilon}\right)_0-\left(\frac{dt}{d\varepsilon}\right)_0\right\}$

As shown using a simple Poisson's ratio calculation in the main text for graphene nanosheet systems, we can state that

$$\left(\frac{dt_c}{d\varepsilon}\right)_0 \gg \left(\frac{dt_x}{d\varepsilon}\right)_0 \tag{S.27}$$

Hence, we can define t_{TNS} as shown.

$$t_{TNS} \approx n_0\left(\frac{dt_c}{d\varepsilon}\right)_0 \tag{S.28}$$

This leads to the much simpler expression below.

$$G \approx G_{TNS} + \frac{t_{TNS}}{t_0 - t_{c,0}} \tag{S.29}$$

Eq.S.29 can also be re-expressed in terms of conductivity,

$$\sigma_0 = \sigma_{Bulk,0} \left(\frac{t_0 - t_{c,0}}{t_{x,0} - t_{c,0}} \right)^{n_0} \tag{S.30}$$

Rewriting the above expression to substitute into Eq.S.29. yields.

$$\frac{1}{t_0 - t_{c,0}} = \frac{1}{t_{x,0} - t_{c,0}} \left(\frac{\sigma_{Bulk,0}}{\sigma_0} \right)^{1/n_0} \tag{S.31}$$

Making the substitution to reintroduce conductivity back into the model.

$$G \approx G_{TNS} + \frac{t_{TNS}}{t_{x,0} - t_{c,0}} \left(\frac{\sigma_{Bulk,0}}{\sigma_0} \right)^{1/n_0} \tag{S.32}$$

Defining σ_{TNS} simplifies the expression

$$\sigma_{TNS} = \sigma_{Bulk,0} \left(\frac{t_{TNS}}{t_{x,0} - t_{c,0}} \right)^{n_0} \tag{S.33}$$

This gives a model relating σ_0 to G.

$$G \approx G_{TNS} + \left[\frac{\sigma_{TNS}}{\sigma_0} \right]^{1/n_0} \tag{S.34}$$

Derivation of $G \propto R_0^{\frac{1}{n+1}}$

From Eq.S.30

$$\sigma_0 = \sigma_{B,0}\left[\frac{t_0 - t_{c,0}}{t_{x,0} - t_{c,0}}\right]^{n_0} \tag{S.35}$$

Provided $t_0 >> t_{c,0}$

$$\sigma_0 \propto t_0^{n_0} \tag{S.36}$$

Writing Resistance in terms of L, t, σ and width (w).

$$R_0 = \frac{L}{\sigma_0 t_0 w} \tag{S.37}$$

$$R_0 \propto \frac{1}{\sigma_0 t_0} \tag{S.38}$$

Substituting in from Eq.S.36

$$R_0 \propto \frac{1}{t_0^{n_0} t_0} \tag{S.39}$$

$$R_0 \propto \frac{1}{t_0^{n_0+1}} \tag{S.40}$$

$$t_0 \propto R_0^{-\frac{1}{n_0+1}} \tag{S.41}$$

From Eq.5 we know

$$G \approx G_{TNS} + \frac{t_{TNS}}{t_0 - t_{c,0}} \tag{S.42}$$

$$G - G_{TNS} \approx \frac{t_{TNS}}{t_0 - t_{c,0}} \tag{S.43}$$

If G_{TNS} and t_{TNS} are small and $t_0 \gg t_{c,0}$ as assumed initially

$$G \propto t_0^{-1} \tag{S.44}$$

Subbing in Eq.S.41 yields the final relation

$$G \propto R_0^{\frac{1}{n+1}} \tag{S.45}$$

Inter- versus intra-nanosheet conduction

It has been argued previously that the conductivity of a nanosheet network scales inversely with $R_{NS} + R_J$, the sum of the resistances of an individual nanosheet and an individual junction.[1] This means

$$\sigma_B = \frac{\alpha}{R_J + R_{NS}} \tag{S.46}$$

where α is a (strain-independent) constant.[2] Differentiating this expression in the limit of low strain yields

$$-\left(\frac{d\ln\sigma_B}{d\varepsilon}\right)_0 = -\frac{1}{\sigma_{B,0}}\left(\frac{d\sigma_B}{d\varepsilon}\right)_0 = \frac{\left(dR_J / d\varepsilon + dR_{NS} / d\varepsilon\right)_0}{R_{J,0} + R_{NS,0}} \tag{S.47}$$

Network Characterisation

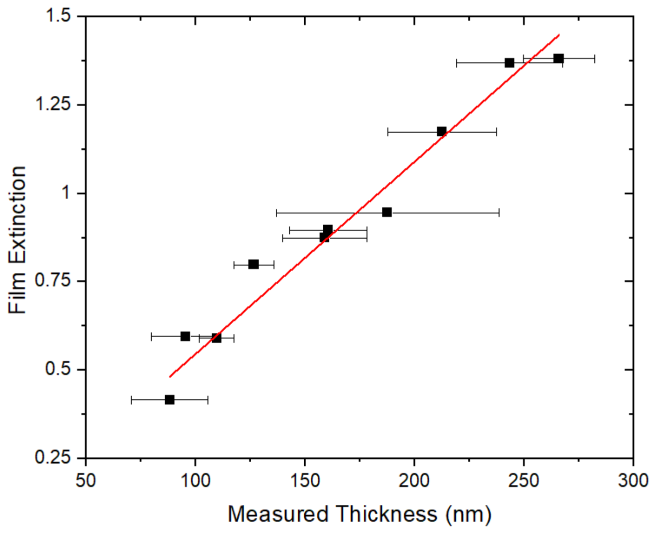

Figure S1: Plot of film extinction Vs. measured thickness for graphene sprayed films on glass substrates showing a linear correlation with a slope of $(54.5 \pm 1.1)\times10^{-4}$ nm^{-1}. Extinction is found from optical transmission measurements while the film thickness was measured by pofilometry. This enabled the thickness of films to be determined using optical measurements.

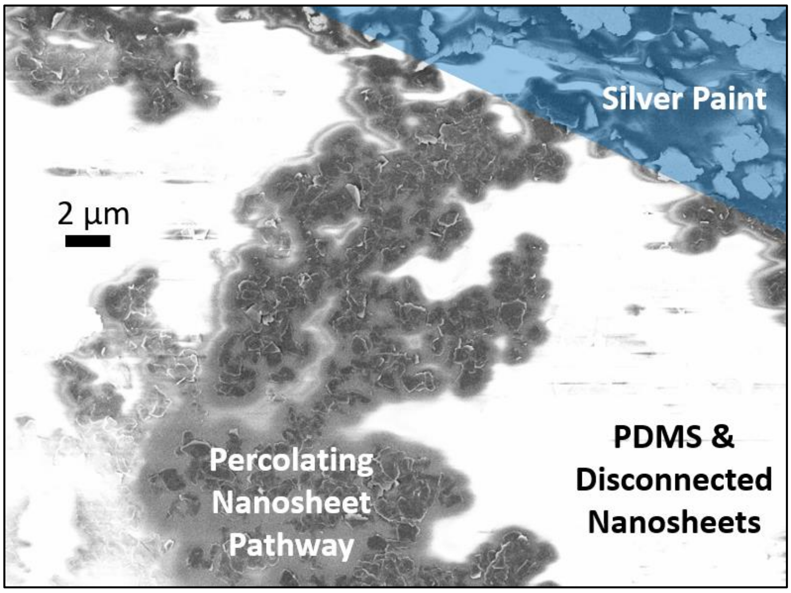

Figure S2: Scanning electron microscope image of a conducting nanosheet pathway contacted by silver paint on one side. Darker regions are the conducting percolative nanosheet pathways through the network. White areas show regions of disconnected nanosheets and uncovered PDMS substrate. The percolative nature of the current carrying pathways, especially in the thinnest networks is illustrated in this image.

Literature Data Comparison

Table S1: Comparison of Gauge Factor and Linear Ranges for a range of graphene only film sensors. *Values extracted from raw data for linear regions.

**Authors**	**Preparation Technique**	**Gauge Factor ***	**Linear Range* (%)**	**Year**	**Reference**
Bae *et al.*	CVD	2.7	1.5%	2013	[3]
Casiraghi *et al.*	Inkjet Printing	125	1.25%	2018	[4]
Hempel *et al.*	Spray Coating	170	1.5%	2012	[5]
Li *et al.*	Drop Casting/Marangoni Self-Assembly	300	0.5%	2016	[6]
Qiao *et al.*	Laser Scribing	54.7	2%	2018	[7]
Tian *et al.*	Laser Scribing	9.49	3%	2014	[8]
Zhao *et al.*	CVD	300	0.3%	2012	[9]
Zhao *et al.*	PECVD	600	1%	2015	[10]

References

(1) Boland, C. S.; Khan, U.; Ryan, G.; Barwich, S.; Charifou, R.; Harvey, A.; Backes, C.; Li, Z.; Ferreira, M. S.; Möbius, M. E.; et al. Sensitive electromechanical sensors using viscoelastic graphene-polymer nanocomposites. *Science* **2016**, *354* (6317), 1257. DOI: 10.1126/science.aag2879.
(2) Kelly, A. G.; O'Suilleabhain, D.; Gabbett, C.; Coleman, J. N. The electrical conductivity of solution-processed nanosheet networks. *Nature Reviews Materials* **2021**. DOI: 10.1038/s41578-021-00386-w.
(3) Bae, S.-H.; Lee, Y.; Sharma, B. K.; Lee, H.-J.; Kim, J.-H.; Ahn, J.-H. Graphene-based transparent strain sensor. *Carbon* **2013**, *51*, 236-242. DOI: 10.1016/j.carbon.2012.08.048.
(4) Casiraghi, C.; Macucci, M.; Parvez, K.; Worsley, R.; Shin, Y.; Bronte, F.; Borri, C.; Paggi, M.; Fiori, G. Inkjet printed 2D-crystal based strain gauges on paper. *Carbon* **2018**, *129*, 462-467. DOI: 10.1016/j.carbon.2017.12.030.
(5) Hempel, M.; Nezich, D.; Kong, J.; Hofmann, M. A novel class of strain gauges based on layered percolative films of 2D materials. *Nano Lett* **2012**, *12* (11), 5714-5718. DOI: 10.1021/nl302959a.
(6) Li, X.; Yang, T.; Yang, Y.; Zhu, J.; Li, L.; Alam, F. E.; Li, X.; Wang, K.; Cheng, H.; Lin, C.-T.; et al. Large-Area Ultrathin Graphene Films by Single-Step Marangoni Self-Assembly for Highly Sensitive Strain Sensing Application. *Advanced Functional Materials* **2016**, *26* (9), 1322-1329. DOI: 10.1002/adfm.201504717.
(7) Qiao, Y.; Wang, Y.; Tian, H.; Li, M.; Jian, J.; Wei, Y.; Tian, Y.; Wang, D. Y.; Pang, Y.; Geng, X.; et al. Multilayer Graphene Epidermal Electronic Skin. *ACS Nano* **2018**, *12* (9), 8839-8846. DOI: 10.1021/acsnano.8b02162.
(8) Tian, H.; Shu, Y.; Cui, Y. L.; Mi, W. T.; Yang, Y.; Xie, D.; Ren, T. L. Scalable fabrication of high-performance and flexible graphene strain sensors. *Nanoscale* **2014**, *6* (2), 699-705. DOI: 10.1039/c3nr04521h.
(9) Zhao, J.; He, C.; Yang, R.; Shi, Z.; Cheng, M.; Yang, W.; Xie, G.; Wang, D.; Shi, D.; Zhang, G. Ultra-sensitive strain sensors based on piezoresistive nanographene films. *Applied Physics Letters* **2012**, *101* (6). DOI: 10.1063/1.4742331.
(10) Zhao, J.; Wang, G.; Yang, R.; Lu, X.; Cheng, M.; He, C.; Xie, G.; Meng, J.; Shi, D.; Zhang, G. Tunable Piezoresistivity of Nanographene Films for Strain Sensing. *ACS Nano* **2015**, *9* (2), 1622-1629. DOI: 10.1021/nn506341u.
